# Study on the Removal of Oxide Scale Formed on 300 M Steel Special-Shaped Hot Forging Surfaces during Heating at Elevated Temperature by a High-Pressure Water Descaling Process

**DOI:** 10.3390/ma16041745

**Published:** 2023-02-20

**Authors:** Fanjiao Gongye, Jie Zhou, Jie Peng, Haicheng Zhang, Shixin Peng, Shishan Li, Heping Deng

**Affiliations:** 1 Chongqing Key Laboratory of Advanced Mold Intelligent Manufacturing, College of Materials Science and Engineering, Chongqing University, Chongqing 400044, China; 2 China National Erzhong Group Deyang Wanhang Die Forging Co., Ltd., Deyang 618013, China

**Keywords:** oxide scale, 300 M steel, special-shaped hot forging, high-pressure water descaling

## Abstract

Numerical simulations and experiments were utilized to study the removal of oxide scale formed on 300 M steel special-shaped hot forging surfaces during heating at elevated temperature by a high-pressure water descaling process. Specifically, the experimental setup of the special-shaped hot forging was designed and manufactured according to the descaling parameters and simulation results obtained from the hot rolling process. The force states of three typical hot forging surfaces impinged by high-pressure water jets were analyzed. Moreover, the mechanism of the high-pressure water descaling process was proposed based on the research results. The numerical simulations and experimental results revealed that the velocity distribution of the high-pressure water jets is relatively different in various areas of the special-shaped hot forging surfaces. Therefore, the descaling performance is synergistically influenced by the velocity of the high-pressure water jet and the shape of the special-shaped hot forging. Given a certain spray pressure, the value of impact force $F_{i}$ plays a significant role in the descaling of the typical hot forging. The larger the value of $F_{i}$ on the typical hot forging surface, the easier it is to remove the oxide scale, and vice versa. Accordingly, the difficulty of removing the oxide scale formed on the 300 M steel special-shaped hot forging surfaces during heating at elevated temperature by a high-pressure water descaling process is in the following order: plane surface < convex surface < concave surface. Additionally, only the inner-layer FeO of the oxide scale remained after the high-pressure water descaling process due to the appearance of FeO-Fe_2_SiO_4_ eutectic in the FeO layer.

## 1. Introduction

Recently, the demand for 300 M steel has significantly increased in aerospace structural components, such as aircraft landing gear and driveshafts, for the advantage it provides in terms of excellent strength, fracture toughness, and fatigue resistance [1,2]. Previous studies have shown that aerospace structural components made of 300 M steel are generally fabricated by high-temperature hot forging followed by machining to further enhance service performance [3,4]. In addition, steel workpieces made of 300 M steel are heated in a furnace before the hot forging operation [5]. Hence, the oxidation phenomenon is inevitable due to the diffusion of cations or anions in the 300 M steel at elevated temperatures [6]. As a result, the oxide scale formed during the heating process can be embedded in the steel workpiece during the forging process, which leads to surface defects of the steel workpiece and wear of the die [5,7]. Therefore, it has commonly been assumed that higher surface quality of the products and longer service lifespan of the die can be obtained if the oxide scale is removed prior to the hot forging operation [8].

For this purpose, it is highly advisable to establish a method to remove the oxide scale in advance of the hot forging operation. Acid pickling [9,10], mechanical descaling [11], abrasive water jet descaling [12,13], and high-pressure water descaling [14] are the methods that are regularly used in industrial fields to remove the oxide scale. Acid pickling has broad applicability, but it induces severe environmental problems due to the emission of nitrogen oxide (NOχ) gases and nitrites. Mechanical descaling is eco-friendly, but it has a low efficiency. Abrasive water jet descaling has a higher impact force than a water jet due to the abrasive in the water, but it is difficult to cover the entire width of the special-shaped hot forging surface with a narrow impact area. In contrast, the high-pressure water descaling process stands out for its broad applicability, high efficiency, and eco-friendliness in the removal of oxide scale formed on 300 M steel special-shaped hot forging surfaces during heating at elevated temperature [15,16,17]. Traditionally, the high-pressure water descaling process is mainly used in hot rolling, and it is widely known that the oxide scales of hot rolling should be removed instantly with the effort of the high-pressure water jet as it impinges on the plane surface [18,19]. Furthermore, there is an increasing number of reports that have studied the high-pressure water descaling process of hot rolling in terms of the nozzle structural parameters, descaling process parameters, and oxide scale formation mechanism [20,21,22,23]. However, the high-pressure water descaling process of special-shaped hot forging is rarely reported. In particular, there has been no general research on the effect of the hot forging shape on the descaling performance when it is subjected to a high-pressure water descaling process. In addition, the mechanism of descaling in the special-shaped hot forging of 300 M steel is rarely discussed. Therefore, it is essential to investigate the high-pressure water descaling process in the special-shaped hot forging of 300 M steel, as well as its mechanism of high-pressure water descaling.

In this study, numerical simulations and experiments were performed to investigate the removal of oxide scale formed on 300 M steel special-shaped hot forging surfaces during heating at elevated temperature by a high-pressure water descaling process; the results are intended to provide substantial guidance for engineering applications.

## 2. Model Theory and Experimental Setup

### 2.1. Analytical Model of Hot Rolling

**Figure 1:**
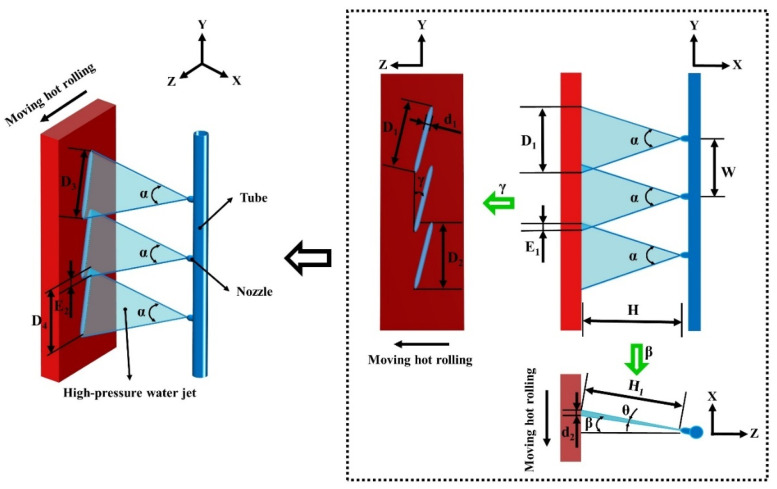
A schematic diagram of the high-pressure water descaling nozzle configuration.

A schematic diagram of the high-pressure water descaling nozzle configuration is shown in Figure 1. A row of nozzles was applied to cover the entire width. The relevant descaling parameters are listed as follows.

As the foremost parameter in the high-pressure water descaling process, the theoretical value of total impact force can be estimated by the following equation [18]:

(1) F=ρ Q ν

where *F* denotes the theoretical value of total impact force (N); ρ is the density of the liquid, expressed here as water (988.2 kg/m^3^); Q is the total flow rate (L/min); and ν is the jet velocity (m/s).

The value of *F* is affected by the following factors: the total flow rate of the nozzle, Q; the velocity of the high-pressure water jet, ν; the spray pressure of the descaling system, P; the spray angle of the nozzle, α; the inclination angle of the nozzle, β; the rotation angle of the nozzle, γ; the jet lateral spray angle of the nozzle, θ; and the spray distance from the nozzle tip to the hot rolling surface, H [24]. Consequently, the actual theoretical value of the total impact force is reduced to

(2) $$F_{t}=0.235Q\sqrt{P}$$

where $F_{t}$ is the actual practical value of the total impact force (N).

Nevertheless, there are certain parameters that need to be considered when configuring high-pressure water descaling nozzles. The descaling process parameters are shown in Figure 1. Additionally, the descaling process parameters can be calculated via the following equations:

(3) $$D_{1}=2H\;tan(\frac{\alpha }{2})$$

where $D_{1}$ is the high-pressure water jet width of the nozzle after a rotation angle of γ (mm) and H is the vertical distance from the nozzle tip to the hot rolling surface (mm), and

(4) $$D_{2}{=Cos\gamma \;D}_{1}$$

where $D_{2}$ is the high-pressure water jet width of the nozzle in the vertical direction of movement along the hot rolling after a rotation angle of γ. Meanwhile, $D_{2}$ is the effective coverage width of descaling (mm).

To obtain better a descaling effect, γ=15° was selected. Besides this, the overlap was selected to be about 10% of $D_{2}$ [25].

(5) $$E_{1}{=D}_{2}\;0.1$$

Here, $E_{1}$ is the overlap of two adjacent high-pressure water jets (mm).

(6) $${W=D}_{1}-{\;E}_{1}$$

Here, W is the distance between two adjacent high-pressure water nozzles (mm).

(7) $$D_{3}=\frac{2H\;tan(\frac{\alpha }{2})}{cos\beta }$$

Here, $D_{3}$ is the high-pressure water jet width of the nozzle after a rotation angle of γ and an inclination angle of β (mm).

(8) $$D_{4}{=Cos\;\gamma \;D}_{3}$$

Here, $D_{4}$ is the high-pressure water jet width of the nozzle in the vertical direction of movement along the hot rolling after a rotation angle of γ and an inclination angle of β (mm).

(9) $${W=D}_{4}-{\;E}_{2}$$

Here, $E_{2}$ is the overlap of two adjacent high-pressure water jets in the vertical direction of movement along the hot rolling after a rotation angle of γ and an inclination angle of β (mm).

(10) $$n=\frac{h+z-E_{2}}{D_{4}-E_{2}}$$

Here, n is the number of nozzles; h is the width of the hot rolling (mm); and z is the allowance of the high-pressure water jet width (mm).

(11) $$A\;\approx {\;d}_{2}{\;D}_{3}$$

Here, $d_{2}$ is the high-pressure water jet thickness of the nozzle after an inclination angle of β (mm).

(12) $$d_{2}\;\approx {\;2H}_{1}\;\tan(\frac{\theta }{2})$$

Here, *A* is the impact area (mm^2^) and $H_{1}$ is the spray distance from the nozzle tip to the hot rolling surface after an inclination angle of β (mm).

(13) $$H_{1}=\frac{H}{cos\beta }$$

(14) $$A\;\approx \;\frac{{4H}^{2}\;tan(\frac{\theta }{2})tan(\frac{\alpha }{2})}{\cos^{2}\beta }$$

(15) $$P_{t}=\frac{F_{t}}{A}$$

Here, $P_{t}$ is the impact pressure (N/mm^2^).

Finally, the actual theoretical value of total impact force can be obtained by the following equation in the case of β=15° [26]:

(16) $$P_{t}=\frac{0.055Q\sqrt{P}}{H^{2}tan(\frac{\theta }{2})tan(\frac{\alpha }{2})}$$

As it can be seen from Equation (16), the values of the descaling process parameters Q, P, α, θ, and H can affect descaling performance via the value of $P_{t}$. More specifically, the value of $P_{t}$ is proportional to the value of Q or P and inversely proportional to the values of α, θ, and H. In contrast, the rotation angle of the nozzle γ aims to avoid collision between two adjacent high-pressure water jets, and it has no direct effect on the value of $P_{t}$.

**Figure 2:**
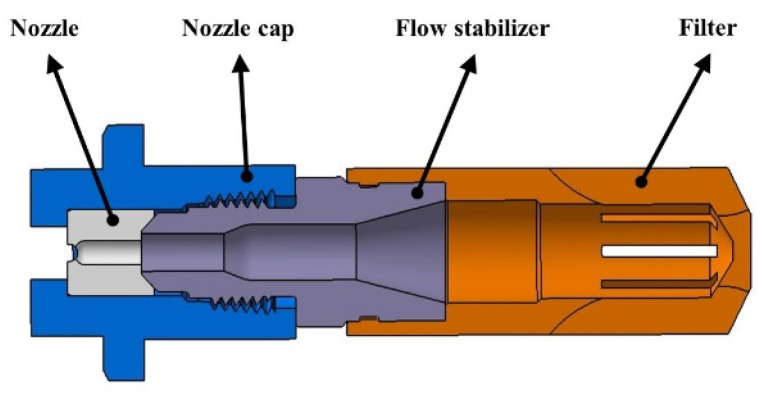
A structural diagram of the high-pressure water descaling nozzle.

Since the nozzle geometry plays a major role in the high-pressure water jet [27], the high-pressure water descaling nozzle selected in this study was the 3212E nozzle manufactured by Spraying Systems Co. with a spray angle of *α* = 35° and a jet lateral spray angle of *θ* = 4°. A structural diagram of the high-pressure water descaling nozzle used in this study is shown in Figure 2. As a flat high-pressure water descaling nozzle, it has four parts, which are the nozzle, nozzle cap, flow stabilizer, and filter.

**Figure 3:**
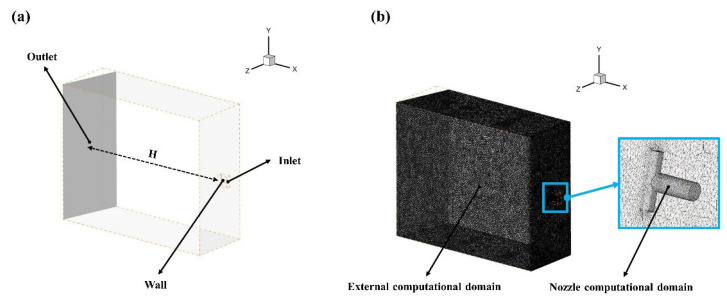
A schematic diagram of the physical model and meshing of hot rolling: (**a**) physical model; (**b**) unstructured grid.

It is widely known that the value of H plays an important role as it can significantly improve the product quality by reducing the spray height [18]. However, excessively reducing the spray height can narrow the impact area, resulting in a reduction in descaling efficiency. Hence, H=105 mm in Figure 3 was selected considering the actual working conditions.

The other descaling process parameters of hot rolling calculated based on Equations (3)–(16) are shown in Table 1.

The three-dimensional modeling software product CATIA V5R21 was utilized to build the physical model. The numerical method was performed using the commercial computational fluid dynamics (CFD) solver FLUENT 2021R2. Figure 3 shows the physical model of hot rolling and its unstructured grid meshing based on the parameters in Table 1. Since all nozzle geometries were equal, only an external computational domain and a nozzle computational domain are shown in Figure 3 in order to save computing resources. The PISO algorithm was used for pressure–velocity coupling. Moreover, the realizable k–epsilon model, scalable wall function model, and Modified Body Force Weighted discretization were applied to perform the numerical simulation.

### 2.2. Analytical Model and Experimental Setup of the Special-Shaped Hot Forging

**Figure 4:**
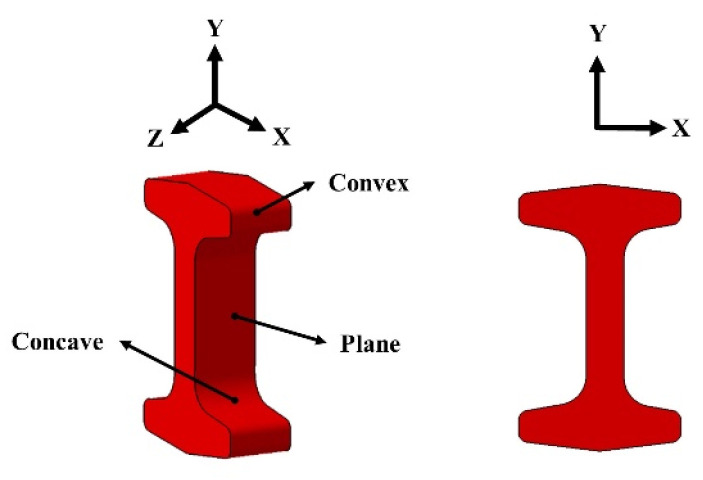
A schematic diagram of the special-shaped hot forging of 300 M steel.

The special-shaped hot forging of 300 M steel with concave, convex, and plane surfaces is presented in Figure 4.

The chemical composition of the as-forged 300 M steel is listed in Table 2.

**Figure 5:**
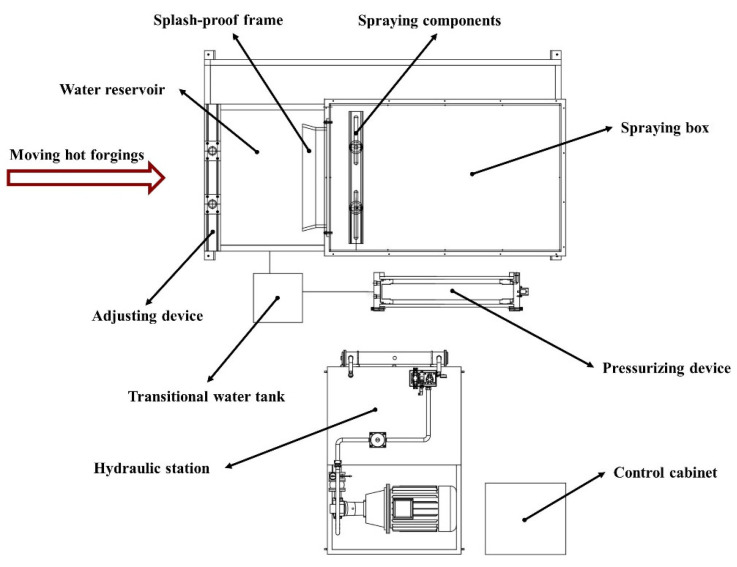
A schematic diagram of the descaling experimental setup.

In this research, the descaling process parameters of hot rolling were applied to the special-shaped hot forging. For this purpose, an experimental apparatus of descaling was designed and manufactured based on the hot rolling process, as shown in Figure 5.

As can be seen in Figure 5, the main parts of the descaling experimental setup are the spraying components, spraying box, pressurizing device, transitional water tank, adjusting device, and water reservoir. It is worth noting that the inclination angle of the nozzle, β, and the rotation angle of the nozzle, γ, can be adjusted depending on the specific shape of the hot forging prior to descaling. Besides this, the spraying components start to work when the hot forging is in the spraying box.

**Figure 6:**
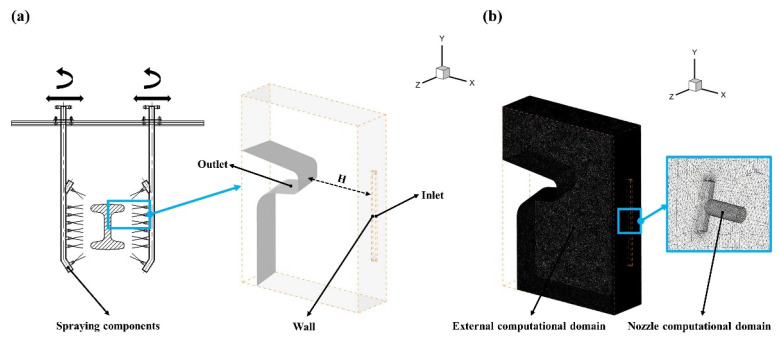
A schematic diagram of the local physical model and meshing of the special-shaped hot forging: (**a**) physical model; (**b**) unstructured grid meshing.

In the present study, the local physical model and meshing of the special-shaped hot forging were applied for the sake of simplicity, as shown in Figure 6. Nevertheless, there was still an external computational domain and three nozzle computational domains to ensure that the high-pressure water jets could cover concave, convex, and plane surfaces. Furthermore, the descaling process parameters and the numerical simulation conditions of the special-shaped hot forging were in accordance with those of the hot rolling.

## 3. Results

### 3.1. Descaling Process of the Hot Rolling

**Figure 7:**
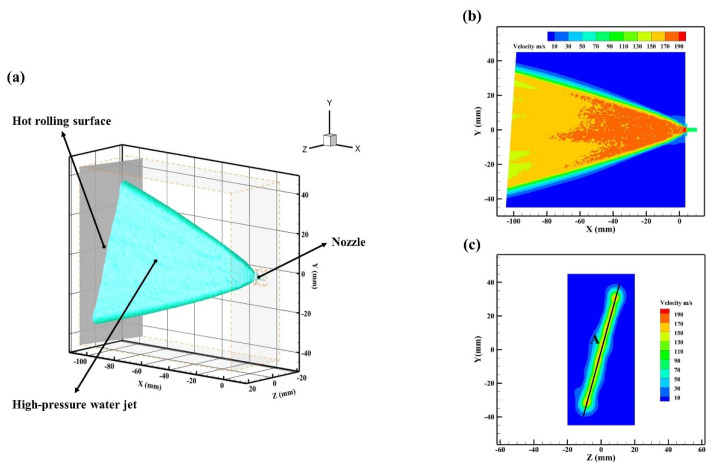
A schematic diagram of simulation results for the hot rolling. (**a**) Iso-surface view of the high-pressure water jet; (**b**) Cross-sectional view of the high-pressure water jet; (**c**) Cross-sectional view of the hot rolling surface.

As can be seen from Figure 7a, the high-pressure water jet was sprayed from the nozzle and impinged on the hot rolling surface when P = 20 MPa. The velocity distributions of the high-pressure water jet cross-section and the hot rolling surface are shown in Figure 7b,c, respectively. The velocity of the high-pressure water jet gradually decreased with increasing spray distance. To be more specific, the velocity of the high-pressure water jet cross-section exceeding 190 m/s was essentially concentrated near the nozzle outlet. In addition, the velocity of the high-pressure water jet on the hot rolling surface was basically over 130 m/s under the condition of H=105 mm.

**Figure 8:**
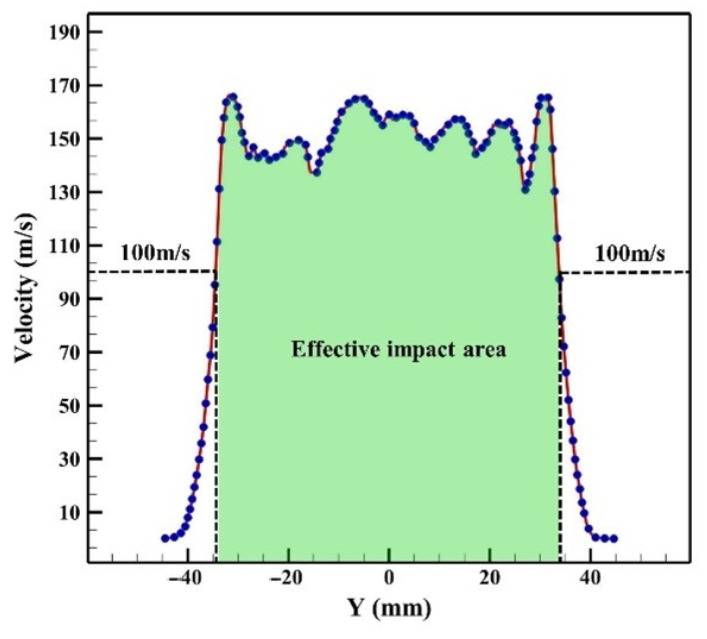
The velocity distribution curve of the high-pressure water jet on the hot rolling surface along line A in Figure 7c.

To provide further analysis of the velocity distribution of the high-pressure water jet on the hot rolling surface, the simulation data of line A in Figure 7c were extracted and are shown in Figure 8.

It is apparent from Figure 8 that the average highest velocity of the high-pressure water jet in the middle of the impact area was around 150 m/s. Furthermore, in the present work, the area over 100 m/s was defined as the effective impact area, which means that the oxide scale in this area could be removed effectively [28]. However, at the edge of the high-pressure water jet area, the velocity dropped dramatically. Nevertheless, the vast majority of the high-pressure water jets had a velocity in excess of 100 m/s in the impact zone.

### 3.2. Descaling Process of the Special-Shaped Hot Forging

**Figure 9:**
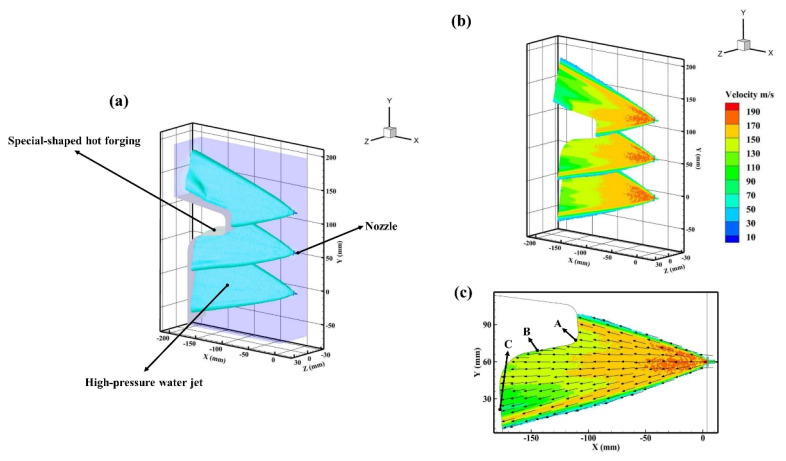
A schematic diagram of simulation results for the special-shaped hot forging. (**a**) Iso-surface view of the high-pressure water jets; (**b**) Cross-sectional view of the high-pressure water jets; (**c**) Cross-sectional view of the high-pressure water jet in the middle.

As can be seen from Figure 9a, the three high-pressure water jets were sprayed simultaneously from the nozzles and impinged on the special-shaped hot forging surface when P = 20 MPa. It is noteworthy that there was no collision between adjacent high-pressure water jets, owing to the existence of the rotation angle of the nozzle, γ. Moreover, the descaling process of the special-shaped hot forging was similar to the descaling process of the hot rolling, except that the surfaces impinged were different. The descaling process of the special-shaped hot forging required impinging not only the plane surface but also the convex and concave surfaces. Hence, the velocity distribution of the high-pressure water jets was relatively different in various areas of the special-shaped hot forging surfaces. We took the middle high-pressure water jet in Figure 9b as an example, as it is the most representative of all three jets. Then, three locations were selected and named as position A, position B, and position C. As shown in Figure 9c, what stands out is that the black arrows in the high-pressure water jet are nearly parallel to the surface at position B, which makes position B relatively difficult to subject to impact force. In comparison, the surfaces at positions A and C are essentially perpendicular to the black arrows, which makes positions A and C more susceptible to the impact force. Therefore, it can be concluded that the surfaces at positions A and C impinged by the high-pressure water jet will outperform the surface at position B in terms of the descaling performance.

**Figure 10:**
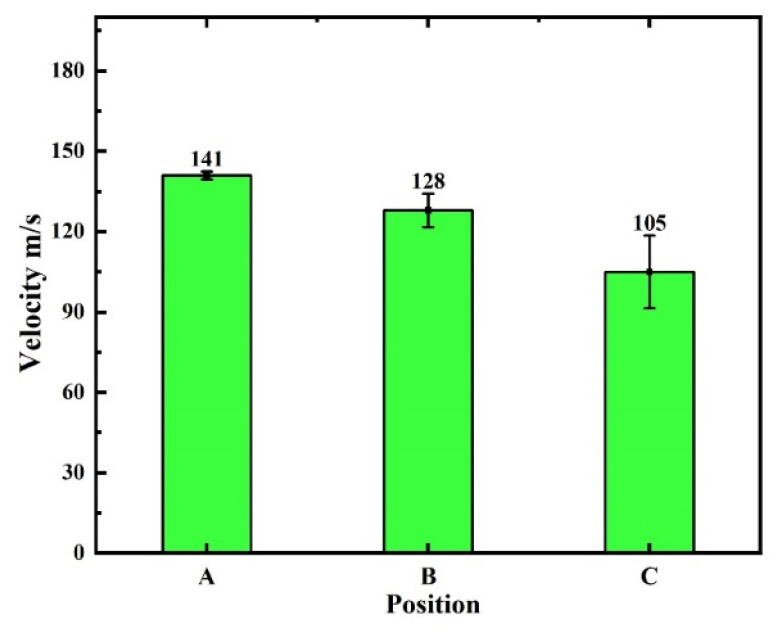
A schematic diagram of the velocity distribution of the high-pressure water jet at positions A, B, and C.

The velocities at position A, position B, and position C in Figure 9c were extracted and are shown in Figure 10. It can be seen from Figure 10 that the velocities at positions A, B, and C were 141 m/s, 128 m/s, and 105 m/s, respectively. It is widely known that the higher the value of velocity, the easier it is to remove the oxide scale from the hot forging surface, and vice versa [29]. However, although the velocity at position B was higher than that at position C, the impact force was dispersed due to its location. As a result, the impact force on the surface of position B was significantly reduced. In addition, position A had the highest velocity compared to positions B and C. Consequently, the difficulty of high-pressure water descaling is in the following order: position A < position C < position B. It is noteworthy that position C is in the effective impact area, which means that the oxide scale on the surface of the special-shaped hot forging can be removed effectively.

**Figure 11:**
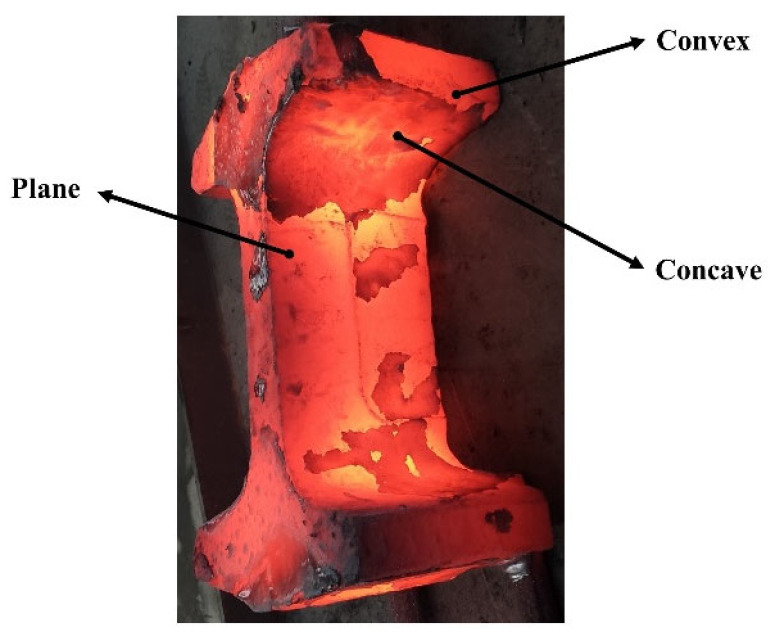
The special-shaped hot forging of 300 M steel after descaling.

In practice, the special-shaped hot forging of 300 M steel was heated at 1200 °C for 1 h before descaling. The descaling result in Figure 11 demonstrates that the high-pressure water descaling process for the special-shaped hot forging of 300 M steel was effective. It can be observed that a large part of the oxide scales that formed on the convex and plane surfaces of the special-shaped hot forging was removed. However, what is striking in Figure 11 is the oxide scale on the concave surface of the special-shaped hot forging, where the oxide scale basically did not peel off. Therefore, the descaling results in Figure 11 are consistent with the conclusion drawn from Figure 9.

## 4. Discussion

### 4.1. High-Pressure Water Jets Impinging on Typical Hot Forging Surfaces

**Figure 12:**
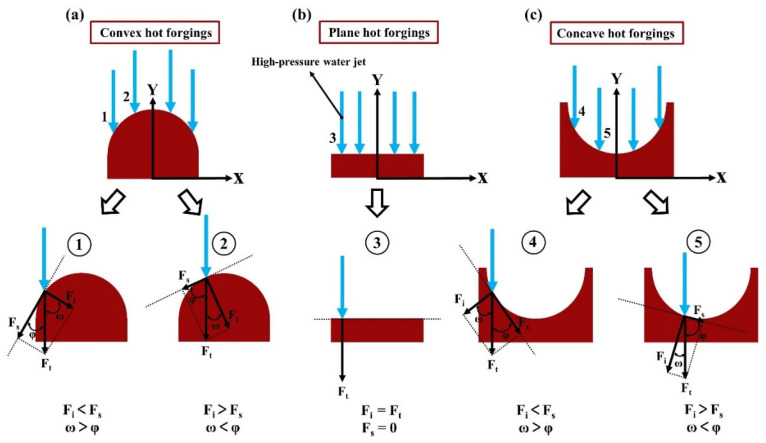
A schematic diagram of force analysis for high-pressure water jets impinging on the typical hot forging surfaces: (**a**) Convex hot forgings; (**b**) Plane hot forgings; (**c**) Concave hot forgings.

To sufficiently make clear the effect of the hot forging shape on the descaling performance when it is subjected to a high-pressure water descaling process, a schematic diagram of high-pressure water jets impinging on the three typical hot forging surfaces was produced, and their force states were analyzed. As shown in Figure 12, high-pressure water jets were simplified as a few blue arrows, and these blue arrows impinge on the convex, plane, and concave surfaces. The spray pressure of the descaling system is generally limited. Therefore, the analysis of the next part was performed given a certain spray pressure.

To start with, when the high-pressure water jets impinge on the typical hot forging surfaces, there exists the total force $F_{t}$, which can be divided into the impact force $F_{i}$ and shear force $F_{s}$. The $F_{i}$ and $F_{s}$ increase with increasing $F_{t}$. Moreover, the angle between $F_{i}$ and $F_{t}$ is defined as *ω,* while the angle between $F_{s}$ and $F_{t}$ is defined as *φ.* The relationship between the values of ω and φ is contrary to that between $F_{i}$ and $F_{s}$. As can be seen from Figure 12a, the total force $F_{t}$ disperses into $F_{i}$ and $F_{s}$ when blue arrow 1 or 2 impinges on a convex surface of a typical hot forging. Blue arrow 1 corresponds to $F_{i}$ < $F_{s}$ and *ω* > φ. Meanwhile, blue arrow 2 corresponds to $F_{i}$ > $F_{s}$ and *ω* < φ. The presence of $F_{i}$ = $F_{t}$ and $F_{s}$ = 0 in Figure 12b when blue arrow 3 is impinging on the plane hot forging is due to the lack of dispersion of the high-pressure water jet. When blue arrows 4 and 5 impinge on the concave surface of hot forging in Figure 12c, blue arrow 4 corresponds to $F_{i}$ < $F_{s}$ and *ω* > φ. Meanwhile, blue arrow 5 corresponds to $F_{i}$ > $F_{s}$ and *ω* < φ. On the one hand, given a certain total force $F_{t}$, $F_{i}$ plays a significant role in the descaling of the typical hot forging. The larger the value of $F_{i}$, the easier it is to remove the oxide scale from the typical hot forging surface, and vice versa. On the other hand, $F_{i}$ and $F_{s}$ are primarily attributed to ω and φ when the high-pressure water jet impinges on the hot forging surface. The values of ω and φ depend on the position where the high-pressure water jet impinges on the surface of the hot forging. More specifically, it is evident that the value of φ is proportional to the value of $F_{i}$, while the value of *ω* is inversely proportional to the value of $F_{i}$. In other words, the value of $F_{i}$ is related to the position where the high-pressure water jet impinges on the typical hot forging surface. The angle between the blue arrow and the tangent line at the impinge position is proportional to $F_{i}$. Therefore, the value of $F_{i}$ increases with increasing value of φ in Figure 12.

The conclusions obtained based on the force analysis in Figure 12 show that the descaling performance is influenced not only by the velocity of the high-pressure water jet but also by the shape of the typical hot forging. As a result, the descaling performance is not directly proportional to the velocity of the high-pressure water jet. Considering a thermal expansion mismatch between the oxide scale and the steel substrate when the high-pressure water jets are impinging on the typical hot forging surface, oxide scale on a convex surface of typical hot forging is easier to remove than that on a concave surface [30]. Besides this, the conclusion above explains why the oxide scales on the convex and plane surfaces of typical special-shaped hot forging were largely peeling off. In contrast, the oxide scales on the concave surface of the typical special-shaped hot forging basically did not peel off, as shown in Figure 11. Accordingly, the difficulty of removing the oxide scale formed on 300 M steel special-shaped hot forging surfaces during heating at elevated temperature by a high-pressure water descaling process is in the following order: plane surface < convex surface < concave surface.

### 4.2. Mechanism of the High-Pressure Water Descaling

**Figure 13:**
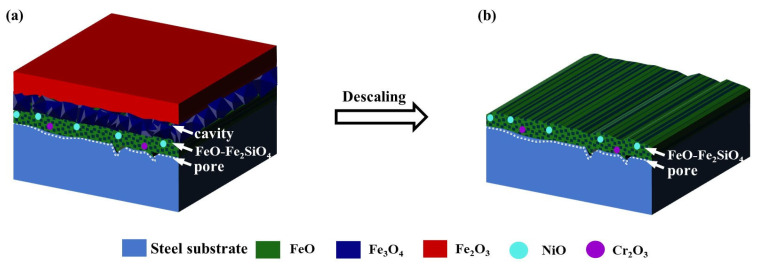
Schematic diagram of the high-pressure water descaling mechanism in 300 M steel at elevated temperature: (**a**) Before descaling; (**b**) After descaling.

The formation mechanism of oxide scale on 300 M steel at elevated temperature was proposed in a previous work [31]. As shown in Figure 13a, there are three layers formed after the oxidation of 300 M steel at elevated temperature: an outer layer of Fe_2_O_3_, an intermediate layer of Fe_3_O_4_, and an inner layer of FeO. It has been reported that the descaling performance is affected by the morphology of the oxide scale, the structure of the oxide scale/steel substrate interface, and cavities in the oxide scale [32]. As a result, only the outer layer Fe_2_O_3_ and intermediate layer Fe_3_O_4_ were removed via a high-pressure water descaling process due to the appearance of molten FeO-Fe_2_SiO_4_ eutectic in the FeO layer formed at 1200 °C [33]. Figure 13b demonstrates that the inner layer of FeO is the remainder of the oxide scale after descaling. In this regard, higher surface quality of the product and a longer service lifespan of the die are obtained due to the fact that the microhardness of the FeO layer is much lower than that of the Fe_2_O_3_ layer.

## 5. Conclusions

An analytical model and experimental setup of a high-pressure water descaling process in the special-shaped hot forging of 300 M steel were designed and manufactured on the basis of the hot rolling process. Then, numerical simulations and experiments were implemented to study the removal of oxide scale formed on 300 M steel special-shaped hot forging surfaces during heating at elevated temperature by a high-pressure water descaling process. The major findings can be summarized as follows:

(1) The analytical model and experimental setup of special-shaped hot forging were successfully designed and manufactured based on the hot rolling process.
(2) The velocity distribution of the high-pressure water jets was relatively different in various areas of the special-shaped hot forging surfaces. Therefore, the descaling performance was influenced not only by the velocity of the high-pressure water jet but also by the shape of the special-shaped hot forging.
(3) Given a certain spray pressure, the value of $F_{i}$ plays a significant role in the descaling of the typical hot forging. Therefore, the larger the value of the impact force $F_{i}$ on the typical hot forging surface, the easier it is to remove the oxide scale, and vice versa. Accordingly, the difficulty of removing the oxide scale formed on 300 M steel special-shaped hot forging surfaces during heating at elevated temperature by a high-pressure water descaling process was in the following order: plane surface < convex surface < concave surface.
(4) Only the outer-layer Fe_2_O_3_ and intermediate-layer Fe_3_O_4_ of the oxide scale were removed via the high-pressure water descaling process due to the appearance of FeO-Fe_2_SiO_4_ eutectic in the FeO layer. The inner-layer FeO of the oxide scale remained after descaling.

## 6. Patent

J. Zhou: F.-J. Gong-Ye, H. Luo. A high-pressure water descaling device and method for forgings: China. ZL 202110377276.1 [P], 8 November 2022 (authorized).

## Figures and Tables

**Table 1:** The descaling process parameters of hot rolling.

*H* (mm)	α (°)	*θ* (°)	β	*γ* (°)	*W* (mm)	*Q* (L/min)	*P* (MPa)
105	35	4	15	15	60	38.2	20

**Table 2:** Chemical composition of the as-forged 300 M steel (wt.%).

C	Si	Cr	Ni	Mn	Mo	S	P	V	Fe
0.40~0.46	1.45~1.80	0.70~0.95	1.65~2.00	0.65~0.90	0.35~0.45	≤0.04	0.035	≤0.05	Bal

## Data Availability

Not applicable.
